# Co–Fe Nanoparticles Wrapped on N-Doped Graphitic Carbons as Highly Selective CO_2_ Methanation Catalysts

**DOI:** 10.1021/acsami.1c05542

**Published:** 2021-07-30

**Authors:** Bogdan Jurca, Lu Peng, Ana Primo, Alvaro Gordillo, Vasile I. Parvulescu, Hermenegildo García

**Affiliations:** † Department of Organic Chemistry and Biochemistry and Catalysis, Faculty of Chemistry, University of Bucharest, Bulevardul Regina Elisabeta 4-12, Bucharest 030016, Romania; ‡ Instituto Universitario de Tecnología Química, Universitat Politècnica de València-Consejo Superior de Investigaciones Científicas, Avenida de los Naranjos s/n, 46022 Valencia, Spain; § BASF SE, 67056 Ludwigshafen am Rhein, Germany

**Keywords:** heterogeneous catalysis, CO_2_ utilization, Sabatier reaction, Co−Fe alloy nanoparticles as catalysts, Co−Fe wrapped on N-doped graphene

## Abstract

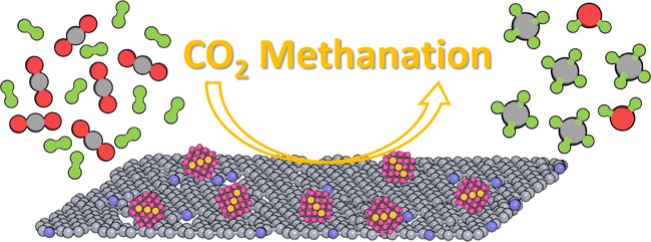

Pyrolysis of chitosan containing various loadings of Co and Fe renders Co–Fe alloy nanoparticles supported on N-doped graphitic carbon. Transmission electron microscopy (TEM) images show that the surface of Co–Fe NPs is partially covered by three or four graphene layers. These Co–Fe@(N)C samples catalyze the Sabatier CO_2_ hydrogenation, increasing the activity and CH_4_ selectivity with the reaction temperature in the range of 300–500 °C. Under optimal conditions, a CH_4_ selectivity of 91% at an 87% CO_2_ conversion was reached at 500 °C and a space velocity of 75 h^–1^ under 10 bar. The Co–Fe alloy nanoparticles supported on N-doped graphitic carbon are remarkably stable and behave differently as an analogous Co–Fe catalyst supported on TiO_2_.

## Introduction

Greenhouse gas emissions are considered responsible for the anthropogenic influence on global warming.^1^ To diminish atmospheric emissions, there is currently much interest in utilizing CO_2_ as feedstock for processes that can be carried out at a very large scale.^2,3^ These new processes should be able to consume very large CO_2_ volumes to make an impact avoiding CO_2_ emissions.^4−6^ Hydrogenation of CO_2_ to obtain hydrocarbons is an exothermic process that can produce useful fuels and chemicals,^7,8^ provided that the H_2_ consumed in the process has a neutral CO_2_ footprint, as the green hydrogen obtained from water electrolysis.^9^ Hydrogenation of CO_2_ to methane, also known as the Sabatier reaction,^10,11^ is one of these possible reactions that can be carried out at a very large scale and has the advantage of making it possible to use the existing natural gas distribution network and infrastructure.

CO_2_ methanation requires active and selective catalysts.^8^ Typical catalysts for this reaction include earth-abundant transition-metal nanoparticles (MNPs), including Ni, Fe, and Cu, supported on large-surface-area inorganic supports.^8,11^ Among these supports, metal oxides are the most commonly used. However, there is an increasing interest in using graphene materials and related large-area graphitic carbons,^12,13^ since the catalytic activity of MNPs supported on defective graphenes may exhibit a unique behavior. It is known that MNPs can anchor on defects and dopant positions, establishing a strong metal–defective graphene interaction that can result in a charge transfer between the graphene sheet and MNPs,^14−16^ tuning the catalytic activity.

In a series of papers, it has been reported that upon pyrolysis of chitosan-adsorbing metal salts, the formation of MNPs supported on N-doped defective graphene sheets and N-doped graphitic carbons can be achieved.^17−19^ The process is straightforward since chitosan and related polysaccharides exhibit a high capacity to adsorb metal salts from water.^20^ In addition, upon pyrolysis, chitosan as a homopolymer of glucosamine acts simultaneously as a source of C and N, resulting in the formation of N-doped, turbostratic graphitic carbons that can be exfoliated to defective graphenes.^21^

Based on these precedents, herein, the preparation from chitosan of Co–Fe alloy NPs wrapped on N-doped graphitic carbon [Co–Fe@(N)C] exhibiting remarkable activity and stability as CO_2_ methanation catalysts is reported. The performance of these Co–Fe alloy NPs on N-doped graphitic carbon improves that of similar Co–Fe alloys supported on TiO_2_, illustrating the advantage of (N)C as a support. Previous studies in the literature have shown the importance of the metal–support interaction and that Co NPs supported on Al_2_O_3_ undergo fast deactivation as catalysts in the Sabatier reaction.^6^

## Results and Discussion

**Scheme 1:**
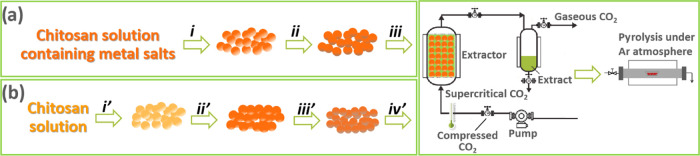
Pictorial Illustration of Preparation Procedures (a) Samples **1**−**3** underwent (i) precipitation in NaOH, and water/EtOH exchange, (ii) NaBH_4_ reduction, and (iii) CO_2_ supercritical drying; (b) Samples **4**−**5** underwent (i′) precipitation in NaOH and water/EtOH exchange, (ii′) metal salt adsorption, (iii′) NaBH_4_ reduction, and (iv′) CO_2_ supercritical drying.

As indicated in Scheme 1, the samples Co–Fe@(N)C under study were prepared by two different procedures. Thus, samples **1**–**3** (Scheme 1a) were prepared in the form of quasi-spherical submillimetric beads by coprecipitation with NaOH of an aqueous chitosan solution acidified by HOAc containing also appropriate amounts of Co(OAc)_2_ and Fe(OAc)_2_. Medium- and high-molecular-weight chitosan is soluble in acidic aqueous solutions but precipitates under neutral or basic conditions. Subsequently, the chitosan spheres containing Co^2+^ and Fe^2+^ salts were dried by a gradual exchange of H_2_O by EtOH and final supercritical CO_2_ extraction of ethanol. This procedure has previously been reported as resulting in highly porous, large-surface-area chitosan aerogels.^22,23^ Final pyrolysis at 900 °C converts the chitosan beads into turbostratic graphitic carbon, accompanied by the simultaneous formation of metallic Co–Fe alloy NPs. Chemical reduction of Co^2+^ and Fe^2+^ ions occurs during the pyrolysis simultaneously with chitosan graphitization due to the reductive conditions of the process derived from the absence of oxygen.^24,25^

In an alternative procedure also indicated in Scheme 1b, Co–Fe@(N)C samples **4** and **5** were prepared by first forming chitosan beads by the NaOH precipitation of the acidic aqueous chitosan solution and then exchanging H_2_O by EtOH before adsorbing Co(OAc)_2_ and Fe(OAc)_2_ from the EtOH solution. Subsequently, Co^2+^ and Fe^2+^ were reduced by NaBH_4_ in EtOH before pyrolysis. For the sake of comparison, one additional sample using TiO_2_ as support was also prepared by wet impregnation of TiO_2_ with an aqueous solution of Co(OAc)_2_ and Fe(OAc)_2_, followed by drying and H_2_ reduction of the resulting powder at 600 °C.

The list of samples, their most important analytical data, and Co–Fe NP size distribution are presented in Table 1, while the Supporting Information (Table S1) indicates the exact amounts of chitosan, Co(OAc)_2_, and Fe(OAc)_2_ used in the preparation of each of the five Co–Fe@(N)C samples.

**Table 1:** Analytical Data and Average Co–Fe Particle Size for the Samples Under Study

sample no.	Co (wt %)^a^	Fe (wt %)^a^	total Co + Fe content (wt %)^a^	C (wt %)^b^	N (wt %)^b^	average particle size (nm)^c^
**1**	4.9		4.9	85.85	1.48	9.5 ± 2
**2**	12.0	5.0	17.0	76.80	1.21	6.9 ± 2
**3**	13.6	5.2	18.8	72.36	0.31	9.7 ± 5
**4**	13.1	4.6	17.7	64.37	0.75	13.3 ± 4
**5**	17.5	4.3	21.8	63.91	1.05	11.2 ± 3

a Determined by ICP-AES analysis after dissolving the metals in aqua regia.

b It is assumed that the rest to 100% is residual oxygen.

c Determined by DF-TEM.

As it can be seen in Table 1, sample **1** contains only Co, while samples **2**–**5** contain a similar Fe content between 4.3 and 5.0 wt %, varying in the Co content from 12.0 to 17.5 wt %. Of note is that the exact metal content of samples **1**–**5** is difficult to predict beforehand in the adsorption step due to the high weight loss resulting in the pyrolysis converting moist chitosan into (N)C. Importantly, samples **1**–**5** contain a residual weight percentage of N from the original chitosan composition that ranges from 0.31 to 1.48 wt % (see Table 1). In previous studies, it has been found that the N content of graphitic carbon derived from chitosan can vary from 6.5 wt % to a negligible value depending on the pyrolysis conditions and the presence of metals that can promote graphitization.^19,26^

X-ray diffraction (XRD) patterns of samples **1**–**5** show that they are constituted by metallic Co and Fe, mainly in the face-centered cubic (fcc) (sample **1**) or body-centered cubic (bcc), accompanied by less intense peaks of the fcc (samples **2**–**5**). No other peaks attributable to metal oxides were recorded in these patterns, indicating that, as expected, Co^2+^ and Fe^2+^ ions have reduced to their metallic state during the pyrolysis process. Previous studies have widely documented that the pyrolysis of carbon precursors results in the chemical reduction of transition metals,^27−30^ including Fe.^31^ To determine if the Co and Fe elements present in the samples are independent Co and Fe particles or if they are alloyed, experimental XRD data were analyzed by Rietveld refinement.

**Figure 1:**
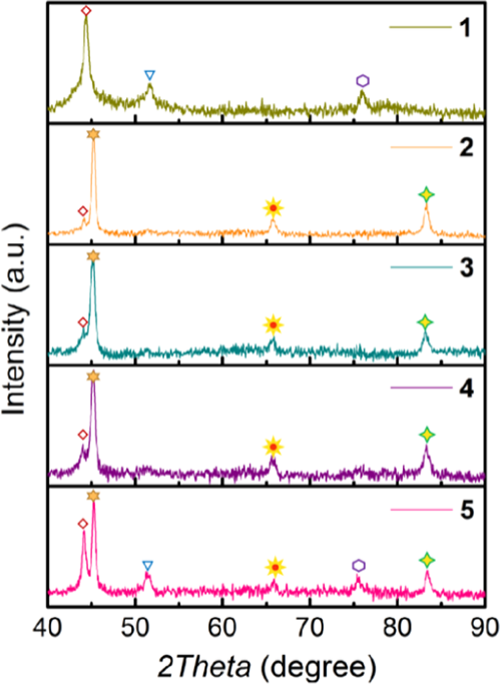
XRD data and the best Rietveld refinement for samples **1**–**5**. Symbols: Co (111) fcc (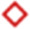); Co (200) fcc (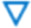); Co (220) fcc (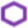); Co (0.7) Fe (0.3) (110) bcc (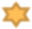); Co (0.7) Fe (0.3) (200) bcc (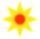); and Co (0.7) Fe (0.3) (211) bcc (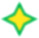).

As an example, Figure 1 shows the fitting of the Rietveld analysis and the experimental data. However, although this XRD analysis supports the formation of Co–Fe alloy, the similarity between the cell parameters of metallic Co and Fe makes necessary additional confirmation by transmission electron microscopy (TEM) to address this issue.

**Figure 2:**
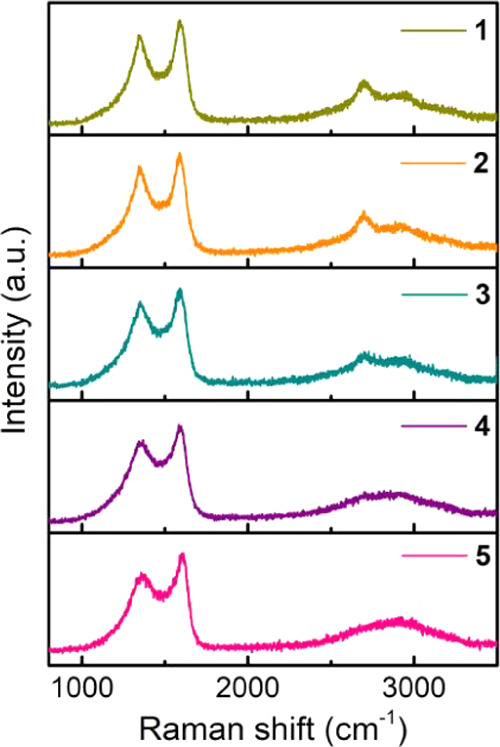
Raman spectrum of samples **1**–**5** recorded upon 514 nm laser excitation.

Transformation of chitosan into N-doped graphitic carbon was assessed by Raman spectroscopy. In the Raman spectra, graphene and graphitic carbons present three characteristic vibration bands at wavenumbers between 3000 and 2600 and between 1590 and 1350 cm^–1^ corresponding to overtones, G and D bands. Figure 2 shows representative Raman spectra for the Co–Fe@(N)C samples under study. The width of the G and D peaks and their relative intensity (*I*_G_/*I*_D_) are taken as quantitative indicators of the quality of the graphene layers. In the present case, the G and D peaks are notably narrower than those previously reported in the pyrolysis of chitosan at 900 °C, probably reflecting the influence of Co–Fe NPs promoting a better graphitization of the (N)C residue. This proposal would be in agreement with the previously commented lower than expected N content of the samples indicated in Table 1. In addition, particularly for samples **1**–**3**, the Raman spectra show a narrow two-dimensional (2D) peak in the high-frequency region at about 2700 cm^–1^. Observation of narrow 2D peaks is associated generally with the presence of few layers of graphene stacking since this 2D peak becomes broader and eventually disappears as the number of stacked graphene layers increases. Thus, Raman spectra indicate that the carbon residue, particularly in samples **1**–**3**, is constituted by the stacking of a few N-doped defective graphene layers.

**Figure 3:**
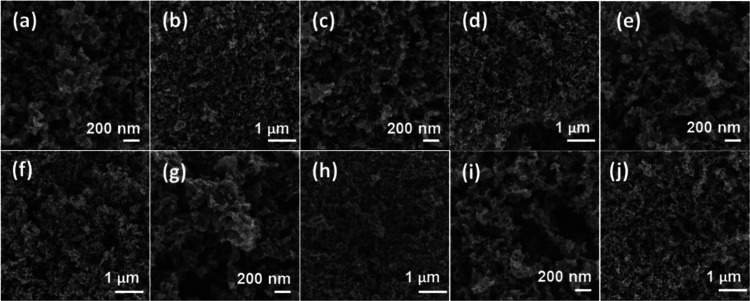
FESEM images of samples **1**–**5**: (a, b) sample **1**; (c, d) sample **2**; (e, f) sample **3**; (g, h) sample **4**; and (i, j) sample **5**.

The morphology of the materials was determined by field emission scanning electron microscopy (FESEM). It seems that the known morphology of chitosan aerogels constituted by the agglomeration of cotton-like, fluffy fibrils is mostly preserved in the pyrolysis during the transformation of chitosan into (N)C residue. Figure 3 shows representative FESEM images of the samples under study in which the loose, coral-shaped, spongy morphology of the samples with considerable macroporosity can be observed. The presence of MNPs could not be visualized by FESEM, meaning that these MNPs should be smaller enough to not become visualized at the 100 nm scale of the technique.

**Figure 4:**
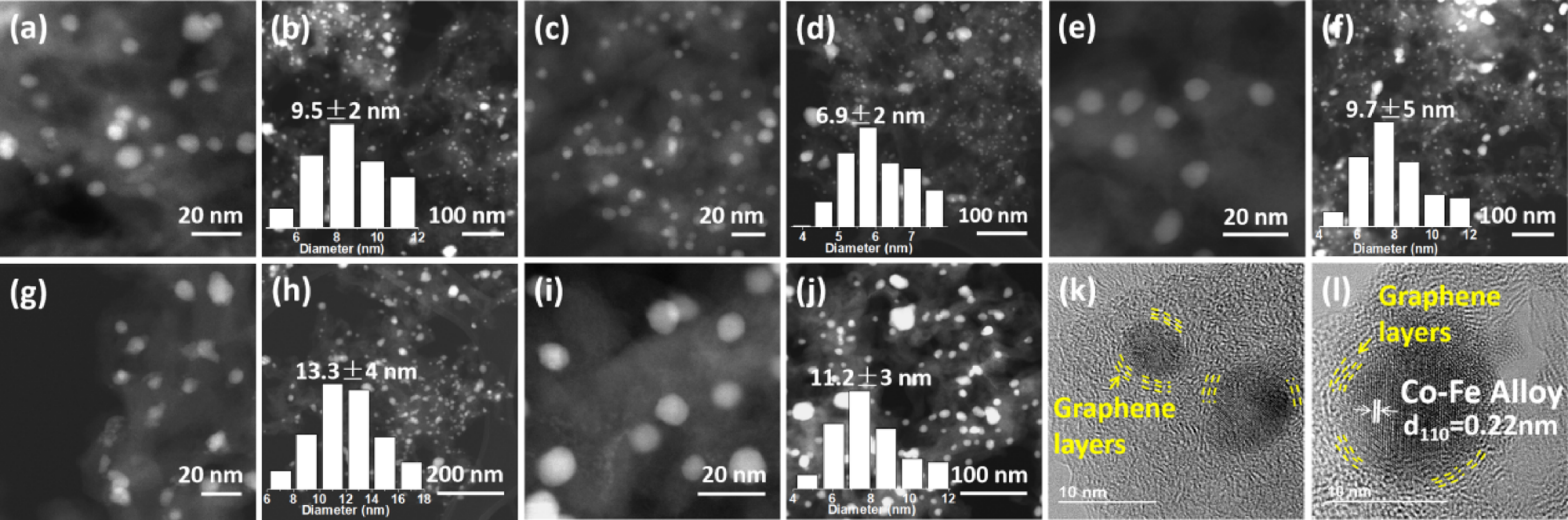
DF-TEM images of samples **1**–**5** and HRTEM images of sample **4**: (a, b) sample **1**; (c, d) sample **2**; (e, f) sample **3**; (g, h, k, l) sample **4**; and (i, j) sample **5**.

The presence of Co–Fe NPs supported on graphene layers of a few micrometers dimension could be clearly detected in the transmission electron microscopy (TEM) images of the samples after ultrasound dispersion of the black carbon powders, both in bright and dark fields (DF). Figure 4 shows selected images of the samples under study, while the Supporting Information (Figure S1) contains a more complete set. The particle size distribution and the average size were determined by measuring a statistically significant number of MNPs. These values are presented in Table 1, while the corresponding histograms are inserted in the TEM images. The average Co–Fe particle size between 6.9 and 13.3 nm with somewhat broad size distribution was estimated from these images. From the high-resolution images, measurement of a fringe distance of 0.22 nm corresponding to the 110 distance of the bcc phase indicates that the Co–Fe NPs correspond to a random alloy since these values are between those corresponding to independent Co and Fe bcc phases.

High-resolution TEM images in bright field also revealed that Co–Fe NPs are wrapped by a few (three and four) layers of N-doped graphene characterized by its typical 0.34 nm interplanar distance. It appears that this wrapping is not complete, but partially covers the surface of the Co–Fe NPs. To illustrate this important point that can serve to understand the role of (N)C on the catalytic activity, Figure 4k,l has marked a representative case, while additional images can be found in the Supporting Information (Figure S2). As commented in the Introduction section, theoretical calculations on models have suggested that graphene can donate charge density to the Co–Fe NP and this charge transfer enriching the electron density at certain atoms of the MNP in contact with the graphene basal plane can act as catalytic sites exhibiting stronger CO_2_ binding.

### Catalytic Activity

The catalytic activity of samples **1**–**5** for CO_2_ methanation was tested in a pressurized fixed-bed, stainless steel reactor in the temperature range of 300–500 °C. Each catalyst was submitted to catalytic tests in which the reactor temperature is increased in 50 °C increments with a dwell time of 1 h. The activity data at each temperature was the average of three analyses of the reaction mixture performed at 30, 45, and 55 min. For all data, the variation among the three analyses was lesser than 5%. Preliminary controls at 500 °C in the absence of any catalyst or using (N)C without any metal as a catalyst showed CO_2_ conversion of 6 and 12.9%, respectively, with methane being the main product with a selectivity over 95%. Previous reports in the literature have shown that N-doped graphene can act as a methanation catalyst,^32^ although the activity measured under our conditions was much lower than that measured for the Co–Fe@(N)C samples.

**Figure 5:**
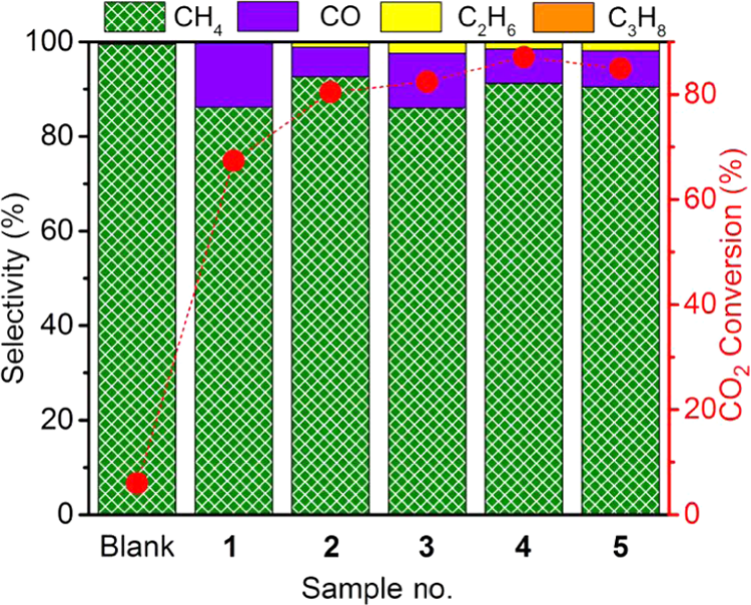
CO_2_ methanation for samples **1**–**5** at 500 °C under the same conditions. (Reaction conditions: H_2_/CO_2_ ratio of 7, total flow of mL/min, 10 bar, 40 mg of catalyst.)

All Co–Fe@(N)C samples exhibit a remarkable catalytic activity for CO_2_ hydrogenation. The main product was CH_4_, accompanied by a lesser percentage of CO. The formation of minute, but detectable, amounts of C_2+_ products constituted by ethane, propane, butane, ethylene, and propylene was also observed. Thermodynamic calculations on CO and CO_2_ hydrogenation, validated in the case of CO, indicate that under the present reaction conditions, the equilibrium should be reached at a very high CO_2_ conversion with 100% selectivity to CH_4_ up to temperatures of 500 °C.^33^ Therefore, although in some cases close to the equilibrium, the data achieved in the present study are not limited by equilibrium considerations. As a general trend of all of the samples, CO_2_ conversion and CH_4_ selectivity increase with temperature, with the highest values in the temperature range under study being measured at 500 °C. Figure 5 summarizes the results obtained for the Co–Fe@(N)C samples under study, while the Supporting Information (Figures S3 and S4 and Tables S2–S7) gathers the full set of data for all of the catalytic studies.

As it can be seen in Figure 5, CO_2_ conversion and CH_4_ selectivity varied also depending on the sample composition. It was observed that sample **1** containing lesser total metal loading reached lower CO_2_ conversions and exhibits higher unwanted CO percentages, compared to the rest of the samples that contain the Co–Fe alloy. On the other hand, the catalytic activity of sample **5** that contained a somewhat higher total metal percentage was lower than that for samples **2**–**4**. Worth noting is the observation of a high percentage of about 20% of C_2+_ products for samples **3** and **4** at 400 °C for CO_2_ conversion above 60%.

**Scheme 2:**
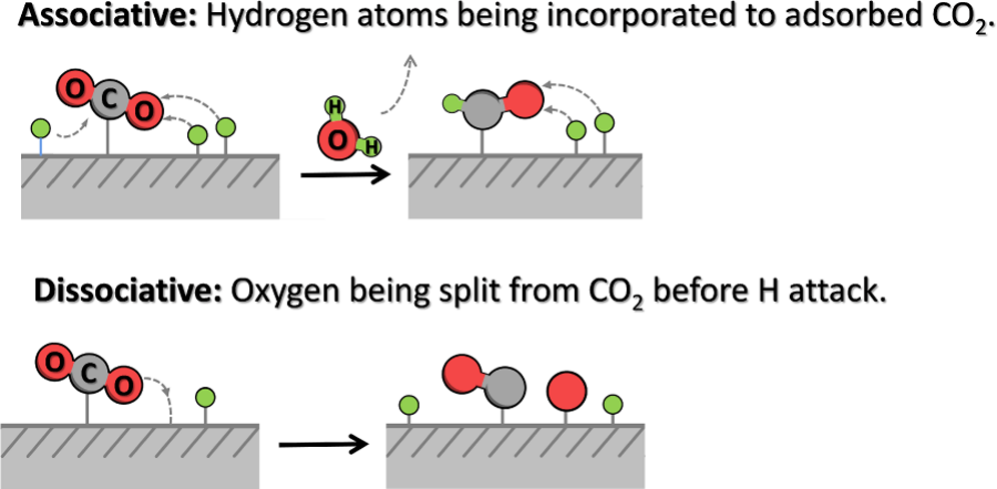
Possible Alternative Pathways Leading to Methanation

It was determined that the best-performing catalyst was sample **4** that reached 87% CO_2_ conversion with a CH_4_ selectivity of 91% operating at 500 °C and a space velocity of 75 h^–1^ at 10 bar. Better performance of sample **4** is observed in spite of the notably larger particle size compared particularly with sample **2** that has a similar composition, but smaller particle size. This different behavior between samples **2** and **4** is most probably due to the different preparation procedures. When the different total metal content of the samples is taken into consideration and turnover frequencies are considered as the figure of merit of the catalytic performance, sample **1** is the best performing due to its low metal content (turnover frequency (TOF) 25.7 s^–1^), while the other samples have similar TOF values of 8.7 s^–1^ for sample **4**, 8.5 s^–1^ for sample **2**, 7.9 s^–1^ for sample **3**, and 7.0 s^–1^ for sample **5**. A plausible reaction mechanism based on the literature for the catalytic CO_2_ methanation on Co–Fe@(N)C is provided in Scheme 2.

The stability of sample **4** was assessed by performing a long-run 30 h experiment at 500 °C observing a constant CO_2_ conversion and product distribution. Then, the sample was screened again for its activity at each temperature between 300 and 500 °C, observing a consistent reproducibility in CO_2_ conversion values and product distribution.

Finally, the influence of (N)C as a support of Co–Fe alloy NPs was assessed by comparing the activity data of samples **1**–**5** with that of Co–Fe NP supported on TiO_2_ (see Table S7). In this case, the main product under the same reaction conditions was CO with a selectivity of 92.8% at a CO_2_ conversion of 29.8% measured at 450 °C. Table S7 contains the full catalytic data for Co–Fe/TiO_2_ as a function of the reaction temperature.

## Conclusions

The present study discloses the two different preparation procedures of Co–Fe alloy NPs supported on N-doped graphitic carbon in which the Co–Fe NPs are partially wrapped by two to four graphene layers. These Co–Fe@(N)C samples exhibit catalytic activity for the Sabatier methanation of CO_2_, reaching CH_4_ selectivity over 90% at high CO_2_ conversion values over 85%. This catalytic activity contrasts with that of similar Co–Fe NPs supported on TiO_2_ for which CO is the main product. These catalysts Co–Fe@(N)C appear to be stable for long-time runs. Overall, the present study shows the potential of chitosan to form graphitic carbon-supported metal catalysts with remarkable activity and stability.

## Supporting Information Available

Experimental section including synthesis of the catalysts, characterization techniques, DF and HRTEM images, methanation activity of samples **1**–**5** and Co–Fe@TiO_2_ at different temperatures (PDF)
