# Supplementary material for: Co–Fe Nanoparticles Wrapped on N-Doped Graphitic Carbons as Highly Selective CO2 Methanation Catalysts
Source: ACS Appl Mater Interfaces. 2021 Jul 30;13(31):36976–81. doi: 10.1021/acsami.1c05542 (PMC9131422; doi:10.1021/acsami.1c05542)
Supplement: Supplementary file 1 — am1c05542_si_001.pdf [file am1c05542_si_001.pdf]

SUPPORTING INFORMATION

Co-Fe Nanoparticles Wrapped on N-Doped Graphitic Carbons as Highly-Selective CO_2 Methanation Catalysts

Bogdan Jurca,[a,⊥] Lu Peng,[b,⊥] Ana Primo,[b] Alvaro Gordillo,[c] Vasile I. Parvulescu[a,] and Hermenegildo García[b,*]*

[a] Department of Organic Chemistry and Biochemistry and catalysis, Faculty of Chemistry, University of Bucharest, Bdul Regina Elisabeta 4-12, Bucharest 030016, Romania.

[b] Instituto Universitario de Tecnología Química, Universitat Politècnica de València-Consejo Superior de Investigaciones Científicas, Av. De los Naranjos s/n, 46022 Valencia, Spain.

[c] BASF SE, 67056 Ludwigshafen am Rhein, Germany

[⊥] Both are first authors.

* vasile.parvulescu@chimie.unibuc.ro (V.P.) and hgarcia@upv.es (H.G.)

Experimental section:

Synthesis of Co@(N)G, Co-Fe@(N)G and Co-Fe@TiO_2

Commercially available reagents were purchased from Aldrich and used without further purification.

Co@(N)G and **Co-Fe@(N)G** were prepared by two different methods. Samples **1-3** were prepared by co-precipitation method. Briefly, 1000 mg chitosan, 625 μL acetic acid and certain amout of $Co(OAc)_2$ and $Fe(OAc)_2$ were added into 50 mL milli-Q water. After chitosan dissolved completely, the solution was introduced dropwise, with a syringe (0.8 mm diameter needle), in an aqueous solution of sodium hydroxide (0.1M). The gel microspheres were formed and immersed in NaOH solution for 2h, then profusely washed with distilled water to pH=7. Then the resulting hydrogel microspheres were washed by a series of ethanol/water baths with an increasing concentration of ethanol (10, 30, 50, 70, 90 and 100 vol%, respectively) for 15 minutes in each. After that, the microspheres were reduced with 500 mL $NaBH_4$-ethanol solution (0.5M) for overnight and exchanged by supercritical CO_2. The resulting microspheres were pyrolyzed under Ar flow (200 mL/min), increasing the temperature at a rate of 2 ºC/min up to 200 ºC for 2h and then to 900 ºC for 2 h. Samples **4** and **5** were prepared were prepared by impregnation of chitosan beads in ethanol solution with iron and cobalt salts. Briefly, 1000 mg chitosan and 625 μL acetic acid were added into 50 mL milli-Q water. After chitosan dissolved completely, the solution was introduced dropwise, with a syringe (0.8 mm diameter needle), in an aqueous solution

of sodium hydroxide (2M). The gel microspheres were formed and immersed in NaOH solution for 2h, then profusely washed with distilled water to pH=7. Then the resulting hydrogel microspheres were washed by a series of ethanol/water baths with an increasing concentration of ethanol (10, 30, 50, 70, 90, 100 vol%, respectively) for 15 minutes in each and immersed in 100 mL Co-Fe-ethanol solution with different concentration for 2d with a slow stirring. After that, the microspheres were reduced with 375 mL $NaBH_4$-ethanol solution with different concentration for a while and then exchanged by supercritical CO_2. The resulting microspheres were pyrolyzed under Ar flow (200 mL/min), increasing the temperature at a rate of 2 ^oC/min up to 200 ^oC for 2h and then to 900 ^oC for 2 h.

Table S1. List of metal source used in the preparation of Co-Fe@(N)C samples **1-5**.

Samples	Metal source	m (mg)
**1**	$Co(OAc)_2$	75
**2**	$Co(OAc)_2$	240
	$Fe(OAc)_3$	100
**3**	$Co(OAc)_2$	240
	$Fe(OAc)_2$	100
**4**	$Co(OAc)_2$	240
	$Fe(OAc)_2$	100
**5**	$CoCl_2$	150
	$FeCl_2$	75

Co-Fe@TiO$_2$: Briefly, to obtain 10 wt% Co and 5% Fe in the final Co-Fe@TiO_2 catalyst, required amount of $Co(OAc)_2$ and $Fe(OAc)_2$ aqueous solution was impregnated to TiO_2 support. After impregnation, the sample was dried at 120 °C for 12h followed by heat treatment in Ar with a heating rate of 2 °C min^{-1} at 400 °C for 4h. Then, the material prepared was reduced by H_2 with a heating rate of 5 °C min^{-1} at 600 °C for 2 h.

Sample characterization

Field Emission scanning electron microscopy (FESEM) images were acquired by using a JEOL JSM 6300 apparatus. HRTEM images were recorded in a JEOL JEM 2100F under an accelerating voltage of 200 kV. Samples were prepared by applying one drop of the suspended material in ethanol onto a carbon-coated nickel TEM grid and allowing them to dry at room temperature. Raman spectra were collected with a Horiba Jobin Yvon-Labram HR UV-visible-NIR (200-1600 nm) Raman Microscope Spectrometer using a 514 nm laser. The chemical composition of the samples was determined by combustion chemical analysis by using a CHNS FISONS elemental analyser.

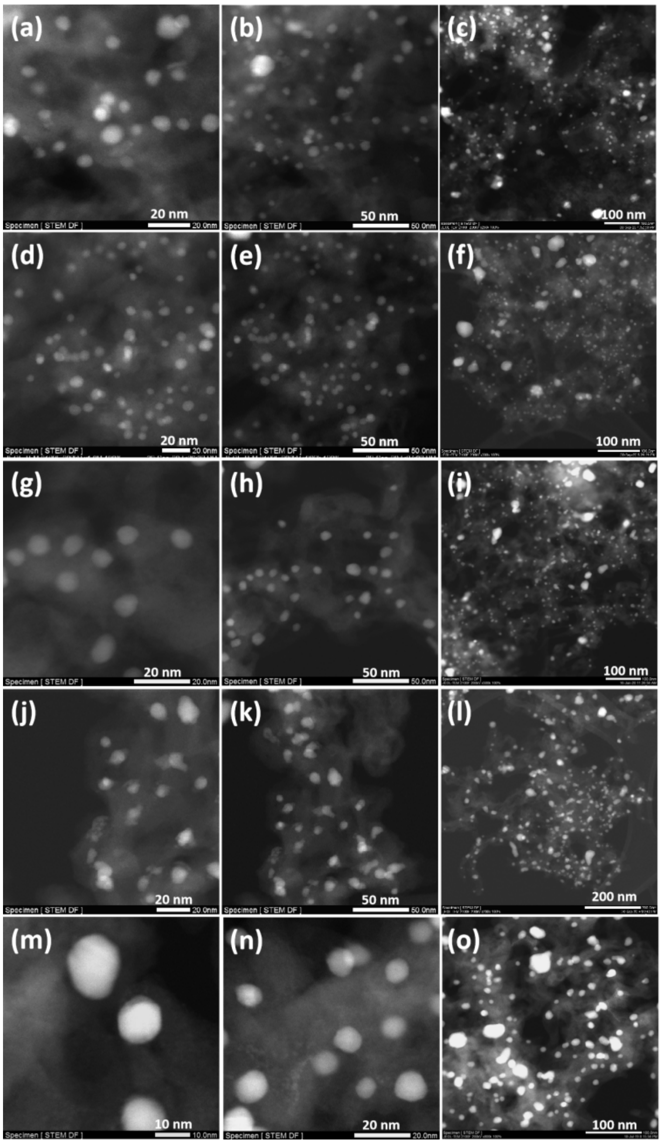

Figure S1. DF-TEM images of samples **1**-**5**. (**a**-**c**: sample **1**; **d**-**f**: sample **2**; **g**-**i**: sample **3**; **j**-**l**: sample **4** and **m**-**o**: sample **5**.)

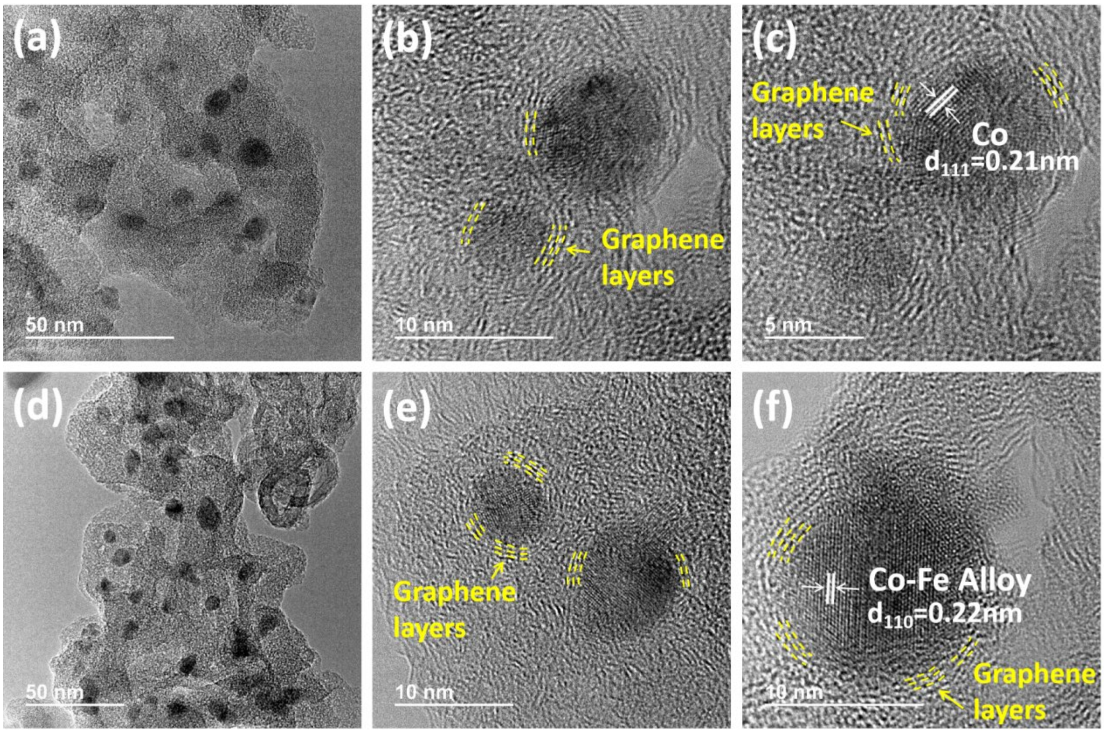

Figure S2. HRTEM images of samples **1** and **4**. (**a-c**: sample **1** and **d-f**: sample **4**.)

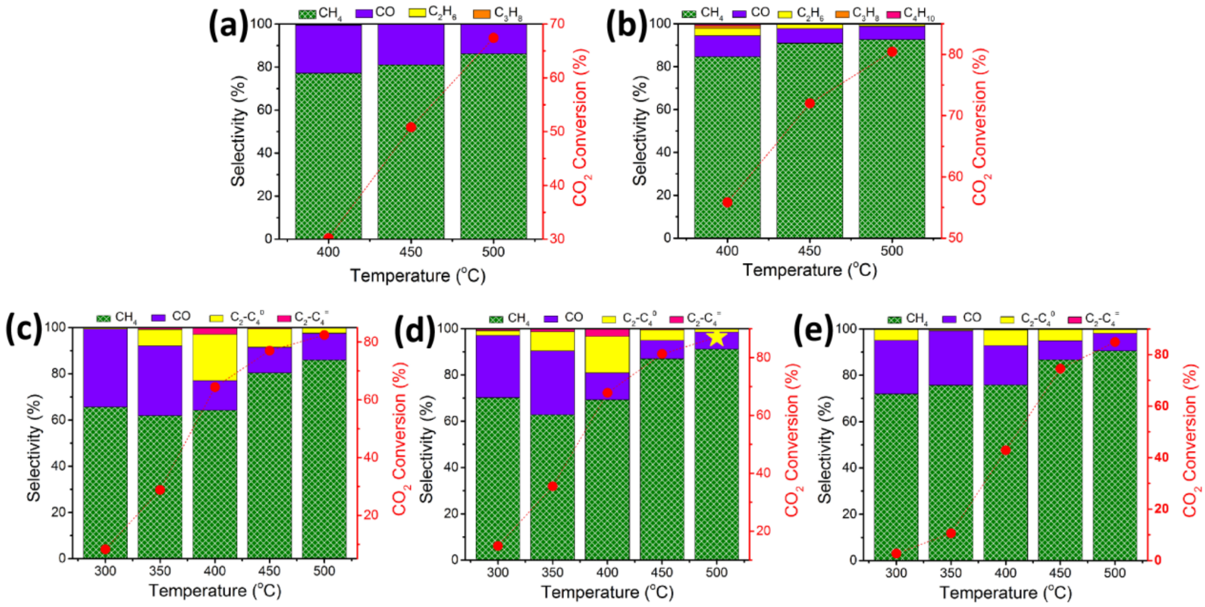

Figure S3. CO_2 methanation of samples **1-5** at different temperatures. (**a**, **b**, **c**, **d**, **e**, corresponding to sample **1-5**, respectively.)

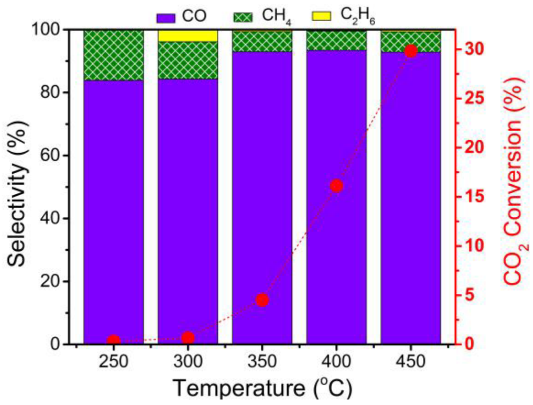

Figure S4 CO_2 conversion and selectivity of Co-Fe@TiO_2 at different temperatures.

Table S2. CO_2 conversion and selectivity for sample **1** at different temperatures. (Reaction conditions: H_2/CO_2 ratio of 7, total flow 4 mL/min, 10 bar, 40 mg catalyst.)

T (°C)	**C (%)**	S (%)	S (%)	S (%)	S (%)	S (%)	S (%)	S (%)
	**CO_2**	CH_4	C_2H_6	C_3H_8	*n*-C_4H_{10}	C_2H_4	C_3H_6	CO
400	**30.2**	77.1	0.3	0.2	0	0	0	22.4
450	**50.8**	81.0	0.2	0	0	0	0	18.8
500	**67.4**	86.2	0.3	0	0	0	0	13.5

Table S3. CO_2 conversion and selectivity for sample **2** at different temperatures. (Reaction conditions: H_2/CO_2 ratio of 7, total flow 4 mL/min, 10 bar, 40 mg catalyst.)

T (°C)	**C (%)**	S (%)	S (%)	S (%)	S (%)	S (%)	S (%)	S (%)
	**CO_2**	CH_4	C_2H_6	C_3H_8	*n*-C_4H_{10}	C_2H_4	C_3H_6	CO
400	**55.9**	84.6	3.4	1.2	1.0	0	0	9.9
450	**72.0**	90.9	2.1	0.2	0	0	0	6.8
500	**80.4**	92.6	1.0	0.1	0	0	0	6.3

Table S4. CO_2 conversion and selectivity for sample **3** at different temperatures. (Reaction conditions: H_2/CO_2 ratio of 7, total flow 4 mL/min, 10 bar, 40 mg catalyst.)

T (ºC)	**C (%) CO_2**	S (%) CH_4	S (%) C_2H_6	S (%) C_3H_8	S (%) *n*-C_4H_{10}	S (%) C_2H_4	S (%) C_3H_6	S (%) CO
300	**8.2**	65.6	0.7	0	0	0	0	33.7
350	**28.8**	61.9	3.8	1.6	1.6	0	0.9	30.3
400	**64.4**	64.2	11.4	5.3	3.4	0.8	2.1	12.8
450	**77.0**	80.3	6.3	1.3	0.4	0.2	0.3	11.2
500	**82.4**	86.0	2.2	0.2	0	0	0	11.6

Table S5. CO_2 conversion and selectivity for sample **4** at different temperatures. (Reaction conditions: H_2/CO_2 ratio of 7, total flow 4 mL/min, 10 bar, 40 mg catalyst.)

T (ºC)	**C (%) CO_2**	S (%) CH_4	S (%) C_2H_6	S (%) C_3H_8	S (%) *n*-C_4H_{10}	S (%) C_2H_4	S (%) C_3H_6	S (%) CO
300	**14.9**	70.1	2.0	0	0	0	0.9	26.9
350	**35.5**	62.8	5.0	1.8	1.5	0	1.3	27.6
400	**67.8**	69.3	9.0	4.6	2.1	1.1	2.2	11.6

450	**81.3**	87.0	3.6	0.7	0.3	0.3	0.2	8.0
500	**87.1**	91.2	1.3	0.2	0	0	0	7.3

Table S6. CO_2 conversion and selectivity for sample **5** at different temperatures. (Reaction conditions: H_2/CO_2 ratio of 7, total flow 4 mL/min, 10 bar, 40 mg catalyst.)

T (°C)	**C (%)**	S (%)	S (%)	S (%)	S (%)	S (%)	S (%)	S (%)
	**CO_2**	CH_4	C_2H_6	C_3H_8	*n*-C_4H_{10}	C_2H_4	C_3H_6	CO
300	**2.7**	71.9	4.9	0	0	0	0	23.1
350	**10.6**	75.7	0.8	0	0	0	0	23.6
400	**42.9**	75.8	4.1	1.6	1.0	0	0.5	17.1
450	**74.6**	86.7	3.8	0.7	0.3	0.1	0.1	8.2
500	**85.0**	90.5	1.7	0.2	0	0	0	7.6

Table S7. CO_2 conversion and selectivity for Co-Fe@TiO_2 at different temperatures. (Reaction conditions: H_2/CO_2 ratio of 7, total flow 4 mL/min, 10 bar, 40 mg catalyst.)

T (°C)	**C (%)**	S (%)	S (%)	S (%)	S (%)	S (%)	S (%)	S (%)
	**CO_2**	CH_4	C_2H_6	C_3H_8	*n*-C_4H_{10}	C_2H_4	C_3H_6	CO

250	**0.3**	16.1	0	0	0	0	0	83.9
300	**0.7**	11.8	3.9	0	0	0	0	84.3
350	**4.5**	6.4	0.7	0	0	0	0	93.0
400	**16.1**	6.1	0.5	0	0	0	0	93.5
450	**29.8**	6.5	0.7	0	0	0	0	92.8
